# Local Translation of Extranuclear Lamin B Promotes Axon Maintenance

**DOI:** 10.1016/j.cell.2011.11.064

**Published:** 2012-02-17

**Authors:** Byung C. Yoon, Hosung Jung, Asha Dwivedy, Catherine M. O'Hare, Krishna H. Zivraj, Christine E. Holt

**Affiliations:** 1 Department of Physiology, Development and Neuroscience, University of Cambridge, Downing Street, Cambridge CB2 3DY, UK

## Abstract

Local protein synthesis plays a key role in regulating stimulus-induced responses in dendrites and axons. Recent genome-wide studies have revealed that thousands of different transcripts reside in these distal neuronal compartments, but identifying those with functionally significant roles presents a challenge. We performed an unbiased screen to look for stimulus-induced, protein synthesis-dependent changes in the proteome **of***Xenopus* retinal ganglion cell (RGC) axons. The intermediate filament protein lamin B2 (LB2), normally associated with the nuclear membrane, was identified as an unexpected major target. Axonal ribosome immunoprecipitation confirmed translation of *lb2* mRNA in vivo. Inhibition of *lb2* mRNA translation in axons in vivo does not affect guidance but causes axonal degeneration. Axonal LB2 associates with mitochondria, and LB2-deficient axons exhibit mitochondrial dysfunction and defects in axonal transport. Our results thus suggest that axonally synthesized lamin B plays a crucial role in axon maintenance by promoting mitochondrial function.

## Graphical abstract

**Graphical Abstract:**

**Figure:**
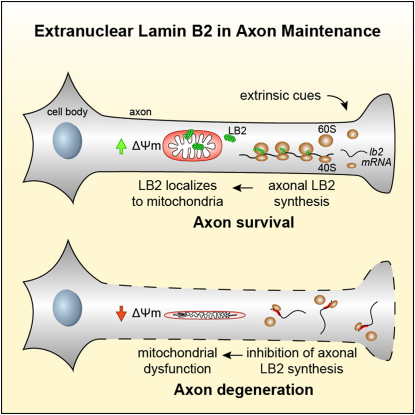

**Highlights:**

► Unbiased proteomics approach to identify cue-induced changes in the axonal proteome ► Lamin B2 is locally synthesized in response to En-1 stimulation in retinal axons ► Loss of axonal lamin B2 leads to axon degeneration independently from the cell body ► Lamin B2 localizes to mitochondria, and its loss leads to mitochondrial dysfunction

## Teaser

An unexpected cytoplasmic role for a lamin protein in axon maintenance could offer insight into the cell type-specific nature of some laminopathies.

## Introduction

Wiring the central nervous system (CNS) requires the precise guidance of axons to distant synaptic targets and their subsequent maintenance. En route, the growth cone (GC) at the tip of a growing axon and remote from the cell body encounters numerous extracellular signals to which it must promptly respond to guide the axon to its synaptic target. Developing axons possess a considerable degree of functional autonomy, and axons severed from their cell bodies can respond to guidance cues in culture (Campbell and Holt, 2001) and navigate accurately in vivo (Harris et al., 1987). This autonomous signal processing in the axon often involves rapid regulation of protein levels in the GC, which is achieved by local protein synthesis and degradation. For example, local translation of *β-actin* (Leung et al., 2006; Yao et al., 2006), *rhoA* (Wu et al., 2005), and *cofiilin* (Piper et al., 2006) mRNAs is required for chemotropic responses of GCs to guidance cues in cultured neurons, and local translation of *ephA2* mRNA is required for midline crossing of chick spinal commissural axons in vivo (Brittis et al., 2002).

An unexplored question is whether axons also draw on local translation for their survival. Distal axons require a constant supply of energy to maintain their integrity, and axonal degeneration is often associated with mitochondrial disorders (Simon and Johns, 1999). One of the first mitochondrial diseases to be understood at a molecular level was Leber's Hereditary Neuropathology (LHON), which results from a mutation in mitochondrial DNA and is characterized by degeneration of the optic nerve (Wallace et al., 1988). Mitochondrial diseases share common pathologies, including axonal degeneration (Pareyson and Marchesi, 2009), with diseases caused by mutations in *lamin* genes, collectively known as laminopathies (Capell and Collins, 2006). Interestingly, *lamin* mRNAs have recently been found in various axons (Gumy et al., 2011; Taylor et al., 2009; Zivraj et al., 2010).

Lamins, encoded by three genes, *lamins A*, *B1*, and *B2*, are type V intermediate filament proteins, which comprise the nuclear lamina inside the inner nuclear membrane. Unlike other types of intermediate filaments such as neurofilaments (type IV) and nestin (type VI), lamins contain a nuclear localization signal (NLS) and in most case a CaaX motif at their C termini. Posttranslational modification of the CaaX motif is responsible for a nonspecific membrane targeting of lamins, which are then imported to the nucleus by a mechanism involving the NLS (Holtz et al., 1989; Loewinger and McKeon, 1988). In addition to providing mechanical support, lamins play critical roles in various nuclear processes such as cell division and transcription (Dechat et al., 2010). Despite their ubiquitous expression in the nuclei of most cells, mutations in *lamin* genes cause a puzzlingly wide array of cell type-specific and subcellular-specific diseases in humans (Capell and Collins, 2006). Although several mutations in *lamin* genes have been associated with nervous system diseases, *lamin A* and *lamin B1* knockout mice have nearly normal nervous systems (Sullivan et al., 1999; Vergnes et al., 2004), suggesting that lamin B2, rather than lamins A and B1, may play a more critical role in the nervous system. Indeed, a recent study showed that *lamin B2* knockout mice have severe defects in the brain resulting from defective neuronal migration during development (Coffinier et al., 2010). Purkinje neurons are particularly affected in the cerebella of these mice (Coffinier et al., 2010), and intriguingly, lamin proteins have been noted in the cytoplasm of these cells (Toesca, 1992), hinting at an extranuclear role for lamins.

Here we show by using a proteomics approach that LB2 is locally synthesized in cultured *Xenopus* retinal ganglion cell (RGC) axons in response to guidance cue stimulation. Accordingly, *lb2* mRNA localizes to and is translated in retinal axons in vivo. Furthermore, LB2 proteins localize to mitochondria in axons, and axonal synthesis of LB2 proteins and their extranuclear actions are required to maintain mitochondrial function and axonal integrity, implicating a role for local translation in preventing axon degeneration. The results show that the functional autonomy of developing axons requires a sustained local supply of LB2 proteins to mitochondria. This mechanism may provide a therapeutic insight into laminopathies by revealing an unexpected link between lamins and axonal mitochondria.

## Results

### Subcellular Profiling using DIGE-NCAT: Identification of Cue-Induced Proteins

**Figure S1:**
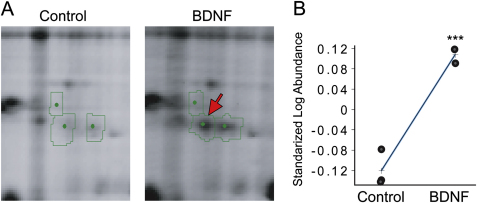
The Level of LB2 Protein in Retinal Cultures Is Increased by BDNF Stimulation, Related to Figure 1 (A) One 2D-DIGE spot (out of ∼1,300 putative spots) that increased in intensity following 24 hr BDNF stimulation of retinal cultures (arrow). (B) Biological variation analysis (BVA) of 2D-DIGE (mean ± SEM; 3 replicates; ^∗∗∗^p < 0.001; unpaired t test). MS analysis identified the spot as LB2 (*Xenopus laevis*; Gene name—*lmnb2*; MASCOT score-66).

**Figure S2:**
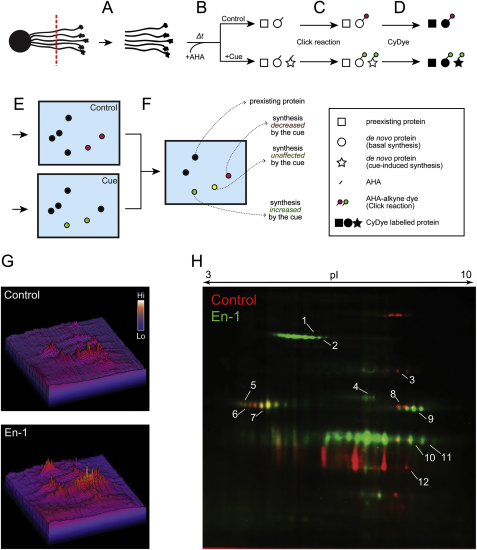
DIGE-NCAT Strategy, Related to Figure 2 (A) The distal portions of the axon bundles in culture were severed from the eyes. (B) The axon-only culture was stimulated with a guidance cue and AHA, and followed by RNA/protein coextraction. Total protein lysate concentrations were matched between conditions based on total RNA concentration. We used RNA concentration because the total amount of axonal protein we could recover was too small for protein-based quantitation. (C) AHA-tagged, newly synthesized polypeptides were reacted with TAMRA-alkyne reporter. (D) TAMRA reacted lysate was mixed with CyDye-labeled standard lysates as well as unlabeled lysates. CyDye-labeled standards were used to normalize multiple gels for analysis, and unlabeled lysates were used to increase the amount of proteins per spot. (E) Combined lysates were separated and visualized by 2D-DIGE. (F) Gels from different conditions were standardized using the CyDye-labeled standard lysate, and spots of interest were picked for MS analysis. (G) 3D representation of DIGE-NCAT spots in control versus En-1 condition with spot intensities represented as peaks. (H) A merged image of AHA-labeled, newly synthesized polypeptides with stimulation of 1 hr control (red) or En-1 (green) shows strikingly different 2D-gel patterns between the two conditions. The numbers indicate the spots picked for MS analysis and correspond to the numbers in Table S1.

Several genome-wide screens using axons/GCs severed from neurons grown in culture have identified thousands of axonally localized mRNAs (Andreassi et al., 2010; Gumy et al., 2011; Taylor et al., 2009; Zivraj et al., 2010)—a puzzlingly large number to provide functionally relevant insights. We sought to identify which of these axonally localized mRNAs are actually translated in response to guidance cues. First, we used 2D difference gel electrophoresis (2D-DIGE), a method that permits quantitative comparative analysis between two proteomes, to ask whether global proteomic differences could be detected after cue stimulation in cultured embryonic *Xenopus* retinal neurons. (Lilley et al., 2002). This method alone, however, was not sufficiently sensitive to detect minute changes in axonal proteomes (Figure S1 available online). Therefore, we developed a DIGE-NCAT (noncanonical amino acid tagging) strategy that combines 2D-DIGE with bioorthogonal noncanonical amino acid tagging (BONCAT) and used it to investigate cue-induced changes in the subcellular proteome of RGC axons. BONCAT uses amino acid analogs (e.g., L-azidohomoalanine: AHA) that are incorporated into de novo polypeptides. These analogs can be linked to a fluorescent tag (e.g., tetramethylrhodamine: TAMRA) by “click chemistry” (Dieterich et al., 2007) and detected after 2D-DIGE. In DIGE-NCAT, axonally synthesized proteins are first labeled with AHA and TAMRA, and the axonal lysates are mixed with CyDye-labeled (used as the internal standard) and unlabeled lysates (used to enrich proteins per spot). The combined lysate is then run on one gel. Using CyDye-labeled spots as reference points, AHA-labeled axonal proteins from different conditions can be compared (Figure S2). Mass spectroscopy (MS) analysis of minute quantities of axonal, AHA-labeled protein is also possible because of retinal lysate enrichment.

**Figure 1:**
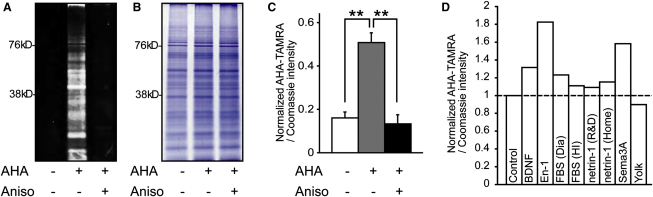
Visualization of Cue-Induced Protein Synthesis (A) Fluorescent 1D gel image showing AHA incorporation in retinal cultures within 1 hr, which is abolished by 40 μM anisomycin. (B) Coomassie staining of the same gel showing similar protein levels in all lanes. (C) Quantitative analysis (mean ± SEM; ^∗∗^p < 0.01; 3 replicates; one-way ANOVA and Bonferroni). (D) AHA incorporation in retinal cultures upon 1 hr stimulation of various cues. See also Figure S1.

We first confirmed that AHA selectively labels newly synthesized polypeptides in retinal cultures in a protein synthesis inhibitor-sensitive manner (Figure 1A). Coomassie staining of the same gel showed that the amounts of total proteins in these groups were indistinguishable (Figure 1B). We used the ratio of AHA to Coomassie intensity as an index of de novo protein synthesis (Figure 1C) and compared axonal protein synthesis induced by different guidance cues (Figure 1D). Engrailed-1 (En-1) elicited the greatest increase (∼80%) (Figure 1D), consistent with previous reports showing that extracellular Engrailed causes rapid translation-dependent guidance responses in RGC axons (Brunet et al., 2005; Wizenmann et al., 2009). Therefore, we chose En-1 as a stimulus to investigate the changes in the axonal proteome in cultured RGC axons.

**Figure 2:**
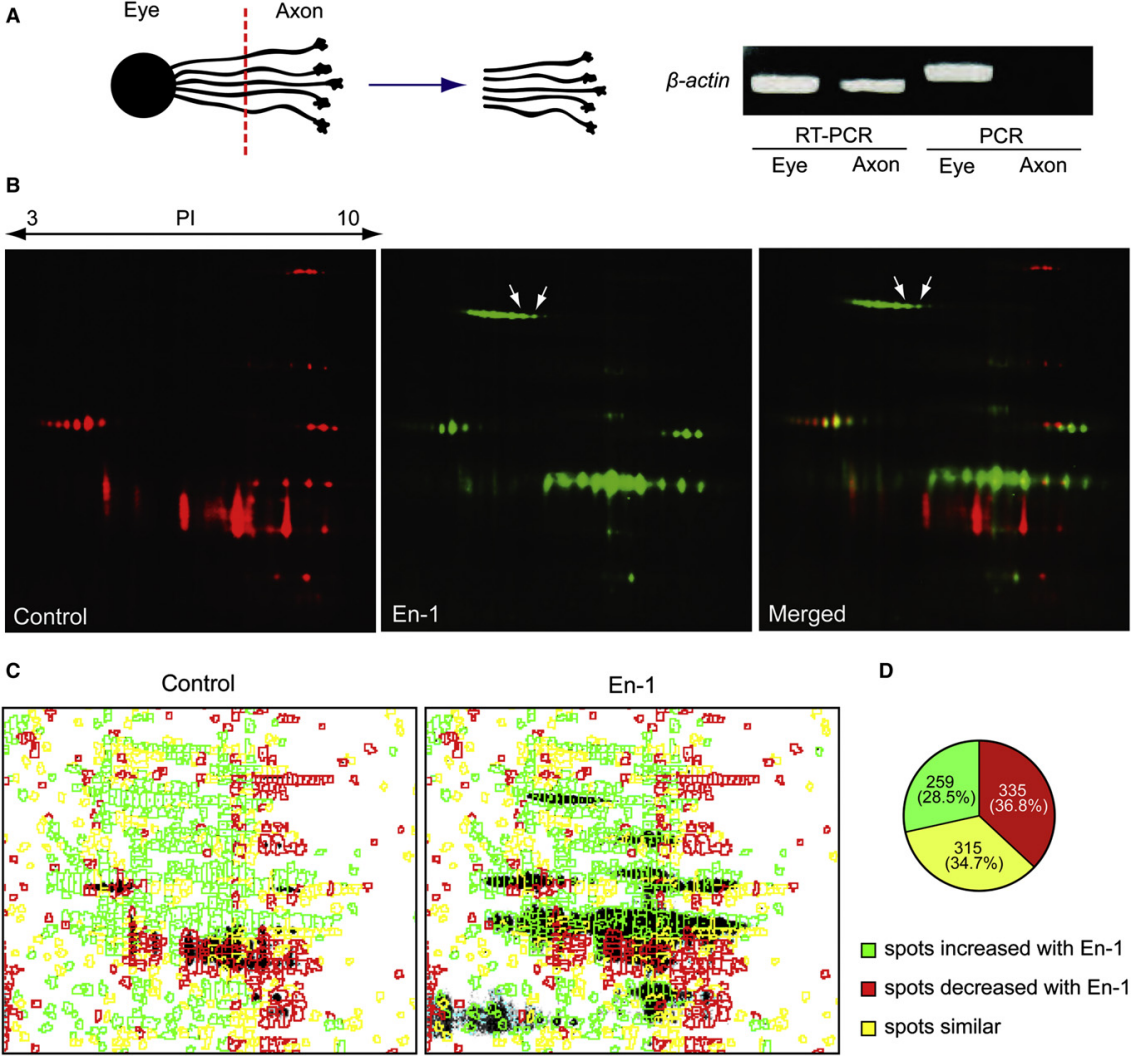
En-1 Stimulation Elicits Dynamic Changes in Local Protein Synthesis (A) Axon culture preparation by separating the eye. The absence of DNA in the axonal fraction confirms its purity. (B) DIGE-NCAT detection of newly synthesized axonal polypeptides shows AHA incorporation in spots corresponding to LB2 (red: control; green: En-1; and arrows: LB2 spot). (C and D) Quantitative analysis of putative protein spots between control and En-1 conditions (green: increased; red: decreased; and yellow: unchanged). See also Figure S2 and Table S1.

To examine specific changes taking place exclusively in the axonal proteome upon En-1 stimulation, we prepared axon-only cultures by culturing whole embryonic eyes, allowing the RGC axons to grow out, and then removing the eyes leaving only the distal axons attached to the substrate (Figure 2A). Typically, 1,000 embryonic eyes were cultured for each condition. PCR amplification of genomic DNA containing the *β-actin* gene in each fraction showed that the axonal fraction was free of cell body contamination (Figure 2A). In contrast, *β-actin* mRNA, which is known to localize to RGC axons in *Xenopus* (Leung et al., 2006), was detected by RT-PCR from the same fraction. These primers flank one intron, and therefore the RT-PCR product is shorter than the genomic DNA PCR product. En-1 stimulation of RGC axons and visualization of newly synthesized proteins with DIGE-NCAT revealed clear differences between control and En-1 conditions (Figure 2B). The overall rate of protein synthesis was significantly increased (Figure S2G), and quantitative analysis revealed that out of 909 putative protein spots detected in the two conditions, 259 (28.5%) spots increased and 335 (36.8%) decreased in intensity by 1.5-fold or more with En-1 stimulation (Figures 2C and 2D). Representative spots from the increased, decreased, or unchanged groups were analyzed by MALDI-TOF MS (Figure S2H and Table S1). The two spots that showed the most robust increase were identified as LB2 (gene name—*lmnb2*; MASCOT scores—254 and 351; Figures 2B, 2C, and S2H; Table S1).

### LB2 Is Locally Synthesized in RGC Axons In Vitro and In Vivo

**Figure 3:**
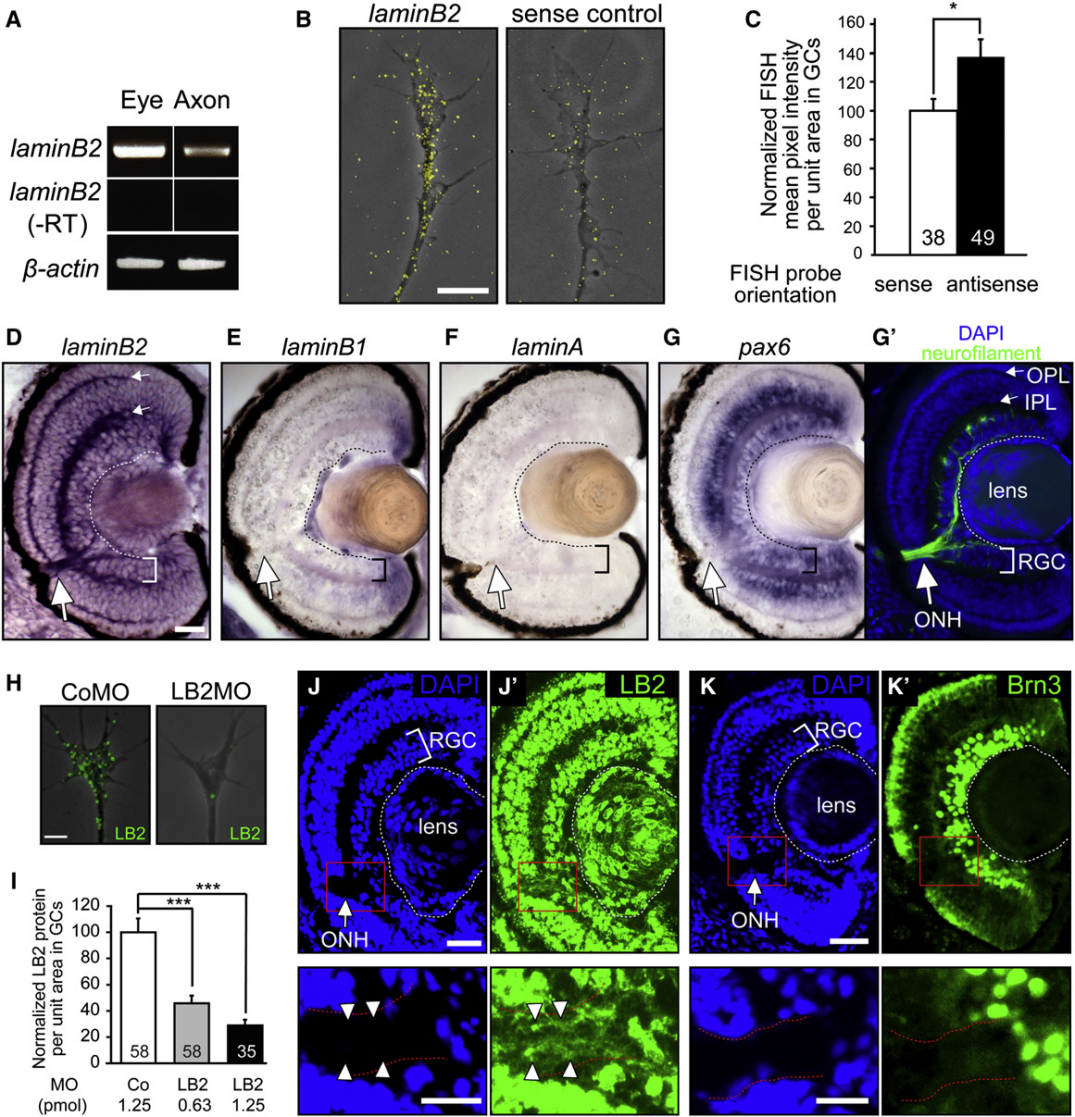
LB2 mRNA and Protein Are Expressed in RGC Axons and GCs (A) Axonal *lb2* mRNA detected by RT-PCR. (B and C) *Lb2* FISH in cultured retinal axons and its quantitation (mean ± SEM; n = no. of GC; ^∗^p < 0.05; Mann-Whitney). (D–G′) ISH of stage 40–45 embryo sections (RGC: retinal ganglion cell layer; ONH: optic nerve head; and IPL/OPL: inner/outer plexiform layer). The same sections were counterstained for neurofilament and DAPI. (H and I) LB2 immunostaining in cultured retinal axons and its reduction by LB2MO. (J and K′) Immunostaining in tissue sections. The boxed areas are shown in the lower panels. Scale bars, 5 μm in (B) and (H), 25 μm in (D)–(G), (J), and (K), and 10 μm in (J) and (K) lower panels. See also Figure S3.

**Figure S3:**
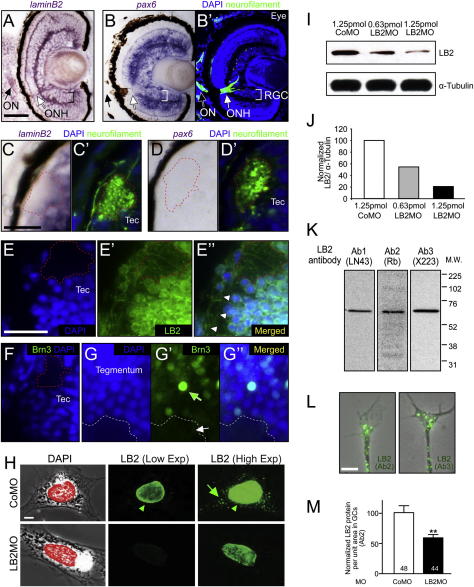
*Lb2* mRNA and LB2 Protein Localize to Axons, Related to Figure 3 (A and B) *Lb2* and *pax6* ISH in eye sections (RGC: retinal ganglion cell layer; ONH: optic nerve head; ON: optic nerve). The same sections were counterstained for axonal (neurofilament) and nuclear (DAPI) markers. The ONH, where RGC axons collect to exit the eye, is devoid of cell bodies and contains *lb2* mRNA but not *pax6* mRNA. (C and D) *Lb2* and *pax6* ISH in brain sections with a counterstained image. The tectal neuropil, where RGC axons terminate, is devoid of cell bodies and contains *lb2* mRNA but not *pax6* mRNA (e.g., inside the red dashed line). (E) LB2 immunostaining in brain sections shows that LB2 localizes to RGC axons in the optic tectum (Tec) (e.g., inside the red dashed line). Arrowheads indicate axons ascending to the optic tectum. (F and G) Brn3, a nuclear factor abundantly expressed in RGCs, does not localize to the tectal neuropil (e.g., inside the red dashed line) or nearby tegmental neuropil (white arrow), although it is highly expressed by a subset of neurons in the tegmentum (green arrow). (H) LB2 antibody recognizes the nucleus (red) as expected (two left panels) in fibroblast-like cells in eye explant culture. Increasing the exposure reveals localization of LB2 outside the nucleus (upper right panel). LB2MO significantly decreases the nuclear LB2 staining (arrowhead), examined at the same exposure (two middle panels). Increasing the exposure shows that the extranuclear LB2 staining (arrow) is also reduced with LB2MO, indicating that the cytoplasmic LB2 signal is specific (two right panels). (I) Western blot of stage 40 embryo head lysate shows that LB2MO inhibits LB2 translation in a dose-dependent manner. (J) Quantification of the lane intensity of LB2/α-tubulin for the indicated conditions. (K and L) Two additional independent antibodies against LB2 detect a single major band in western blot and extranuclear LB2 in axons and GCs in culture. (M) LB2MO reduces axonal LB2 detected by a different antibody confirming the specificity of this signal (^∗∗^p < 0.01; unpaired t test). Scale bars: 25 μm in (A)–(E), 5 μm in (F) and (J).

Identification of LB2 synthesis in axons was unexpected because lamins are major constituents of the nuclear envelope (Hutchison, 2002), and thus our results raised the intriguing possibility of an extranuclear role for locally synthesized LB2 in neurons. If LB2 were axonally synthesized, its transcripts and proteins would be expected to reside in the axons. Using RT-PCR and fluorescent in situ hybridization (FISH), we determined that *lb2* transcripts localize to retinal axons and GCs in vitro (Figures 3A–3C). In situ hybridization (ISH) of retinal sections showed strong staining in the optic nerve head (ONH), a nucleus-free, axon-only structure where the RGC axons collect to exit the eye (Figure 3G′), indicating that *lb2* mRNA localizes to axons in vivo (Figure 3D). Abundant signal was also observed in the inner and outer plexiform layers (IPL and OPL, respectively), suggestive of localization in the dendrites of RGCs and in the axons of other retinal neurons such as bipolar cells. RGCs express little or no mRNAs encoding other lamins (A and B1) (Figures 3E and 3F). In sharp contrast to *lb2*, *pax6* mRNA, which encodes a nuclear protein also highly expressed by RGCs, does not localize to the ONH (Figure 3G), suggesting that specific axonal transport rather than diffusion accounts for the axonal localization of *lb2* mRNA. Axonal *lb2* mRNA is also detected outside the eye, including the neuropil of the optic tectum where RGC axons terminate (Figures S3A–S3D). To examine the expression of LB2 protein, we used a specific antibody against LB2, which detected a single band of the predicted molecular weight on a western blot (Figure S3K) and a specific nuclear signal in immunocytochemistry (Figure S3H). Immunostaining revealed LB2 expression in axons and GCs in vitro (Figure 3H), which showed a dose-dependent reduction when *lb2* mRNA translation was inhibited with a translation-blocking antisense morpholino (LB2MO), verifying specificity (Figures 3H, 3I, S3I, and S3J). Experiments with two additional antibodies, which specifically recognize LB2, confirmed this finding (Figures S3K–S3M). Axonal LB2 protein was also detected in vivo in the ONH, optic nerve, and tectal neuropil (Figures 3J, 3K, and S3E–S3G). Of note, fibroblast-like cells in culture exhibited specific LB2 immunostaining in the cytoplasm in addition to a strong nuclear signal (Figure S3H), suggesting that the extranuclear localization of LB2 is not limited to RGCs.

**Figure 4:**
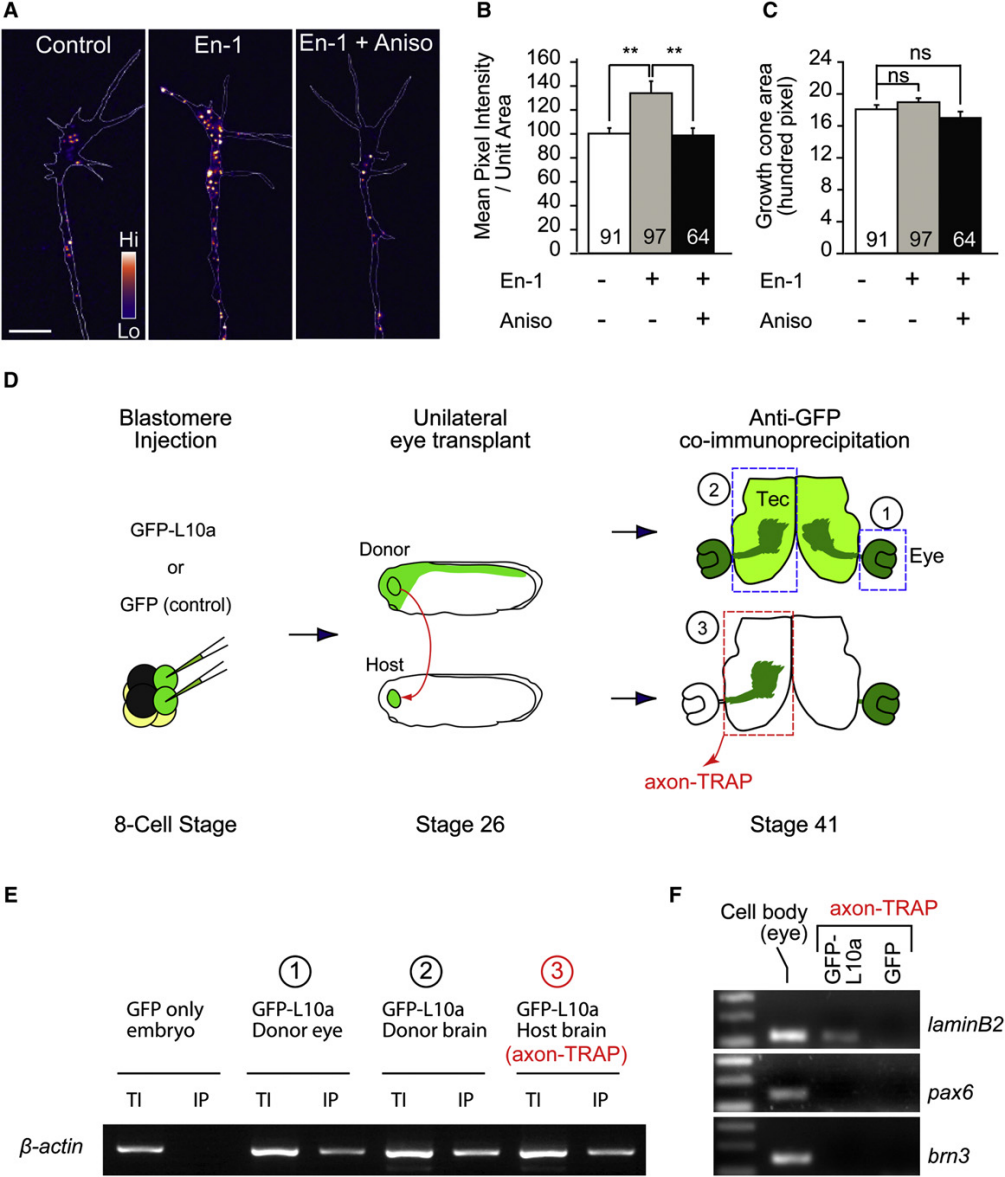
LB2 Is Locally Synthesized In Vitro and In Vivo (A–C) LB2 QIF from axon-only culture (mean ± SEM; n = no. of GCs; 3 replicates; ^∗∗^p < 0.01; one-way ANOVA and Bonferroni). (D) Axon-TRAP experiment. *GFP-L10a RNA* is expressed by blastomere injections in the CNS of a donor embryo, whose eye is transplanted into an uninjected host. The transplanted eye then extends retinal axons to the contralateral optic tectum of the host brain. The third diagram represents a brain that has been cut at the ventral midline and flattened. The boxed areas were dissected out, from which GFP-L10a-containing ribosomes (green) and associated mRNAs were purified by GFP immunoprecipitation. In the negative control, *GFP RNA* was used instead of *L10a-GFP RNA*. (E) RT-PCR from axon-TRAP for *β-actin* mRNA (TI: total input; IP: immunoprecipitation). (F) RT-PCR for *lb2* mRNA. Scale bar, 4 μm. See also Figure S4.

**Figure S4:**
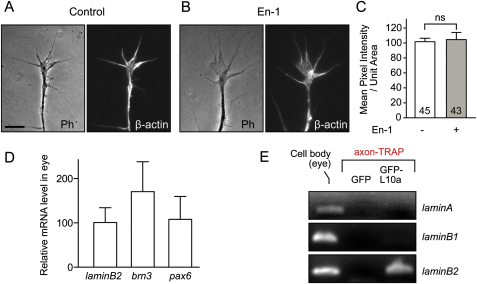
Axonal Translation of *lb2* mRNA Is Selectively Regulated, Related to Figure 4 (A–C) One hour En-1 stimulation does not induce translation of β-actin mRNA, which is localized to RGC axons. ns: not significant; unpaired t test. (D) Lb2 mRNA is expressed at a similar level in the eye to other mRNAs encoding nuclear proteins as revealed by quantitatve RT-PCR. (E) Axon-TRAP analysis shows that other lamin mRNAs are not associated with ribosomes in RGC axons in vivo. Scale bar, 5 μm.

We next used a different method, quantitative immunofluorescence (QIF), to examine En-1-induced LB2 synthesis in axons. Indeed, 1 hr stimulation with En-1 resulted in a 34% translation-dependent increase of LB2 proteins in GCs severed from their cell bodies (Figures 4A and 4B). This increase was not a secondary result of differences in GC morphology, as the GC area was not affected (Figure 4C). In contrast, En-1 did not stimulate the axonal translation of *β-actin* mRNA, which localizes to these GCs (Leung et al., 2006) (Figures S4A–S4C). Next, we investigated whether *lb2* mRNA is axonally translated in vivo. We employed translating ribosome affinity purification (TRAP), which enables isolation of actively translating mRNAs (i.e., mRNAs bound to ribosomes) by expressing a GFP-tagged ribosomal protein, L10a, in cells of interest (Heiman et al., 2008). To examine LB2 synthesis exclusively in retinal axons, we transplanted GFP-L10a-expressing eye primordia to unlabeled hosts (Figure 4D). The grafted eye sent out GFP-L10a-expressing RGC axons to the contralateral optic tectum of the host embryo, and the host brains containing the GFP-L10a-expressing RGC axons were collected (without eyes) and affinity purified against GFP. Because RGCs are the only neurons sending a projection out of the eye and into the brain, this enabled us to examine mRNAs associated with GFP-L10a (i.e., ribosomes) exclusively from developing RGC axons in vivo (axon-TRAP). RT-PCR from axon-TRAP confirmed the presence of *β-actin* mRNA—a molecule known to be locally synthesized in RGC axons (Leung et al., 2006)—and *lb2* mRNA in the axonal translatome (Figures 4E and 4F). In contrast, *pax6* and *brn3* mRNAs, which encode nuclear proteins and are expressed in the eyes at similar levels to *lb2* mRNA, were not present (Figure S4D). *Lamin A* and *B1* mRNAs were not isolated by axon-TRAP (Figure S4E), as expected from their negligible levels of expression in RGCs (Figures 3E and 3F). These results suggest that *lb2* mRNA is indeed locally translated in RGC axons in vivo.

### Local Synthesis of LB2 Is Critical for Axon Survival In Vivo

**Figure 5:**
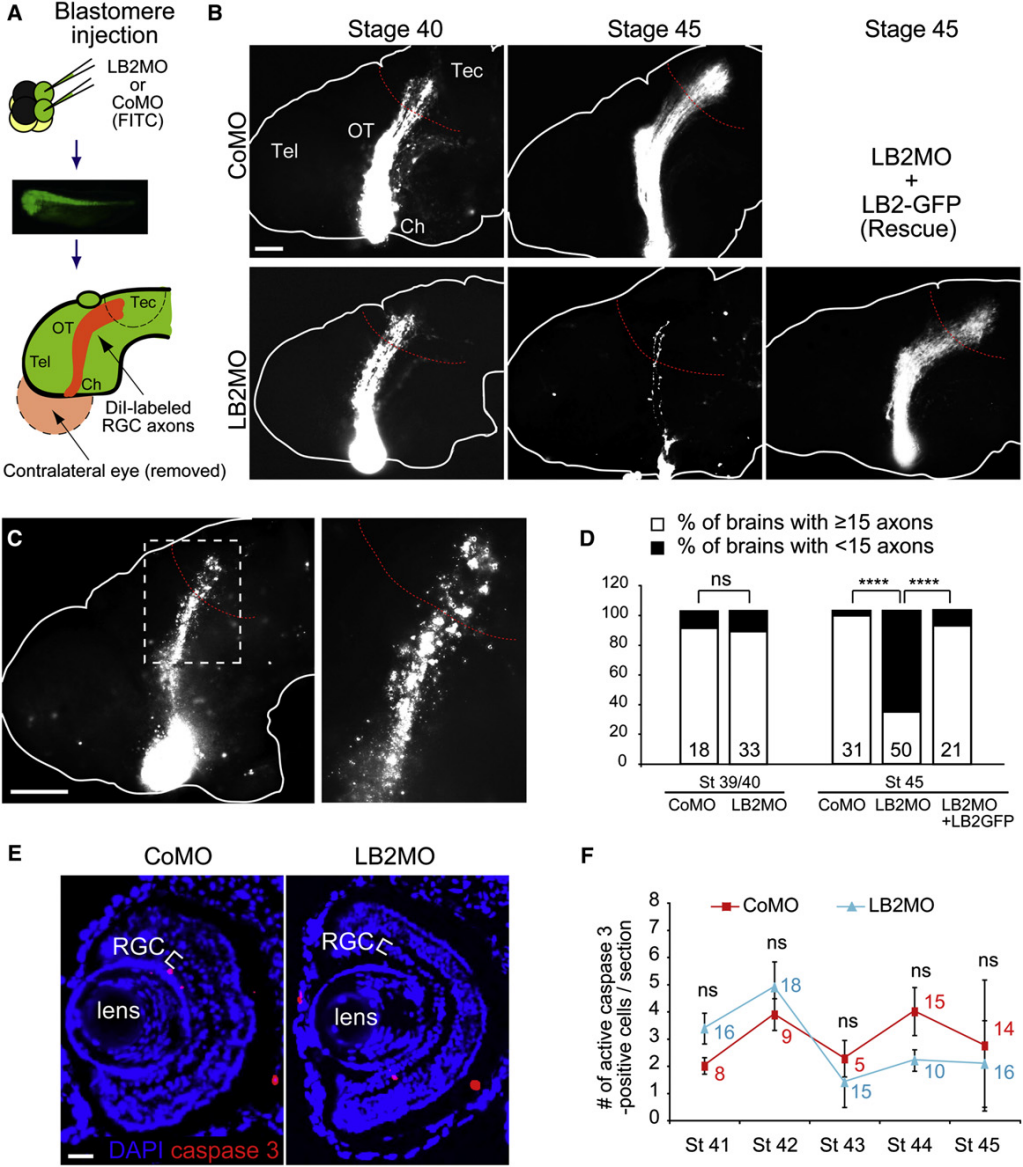
Inhibiting LB2 Translation Results in Degeneration of RGC Pathway In Vivo (A) MO injection and RGC axon labeling using DiI (green: MO; Tel: telencephalon; Ch: optic chiasm; OT: optic tract; Tec: optic tectum; and red: DiI). (B) DiI-labeled RGC axons in MO-injected embryos with or without LB2-GFP RNA. (C) Characteristic beaded morphology of dying axons in LB2 morphants. The boxed area is shown in the right panel. (D) Quantitative analysis of the LB2MO-induced reduction in RGC axons and its rescue by LB2-GFP (n = no. of brains; 3 replicates; ^∗∗∗∗^p < 0.0001; Fisher's exact). (E and F) The frequency of active caspase-3-positive cells in retinal sections (mean ± SEM; n = sections analyzed; Mann-Whitney). Scale bars: 65 μm in (B) and (C), 100 μm in (C) right panel, and 25 μm in (E). See also Figure S5.

**Figure S5:**
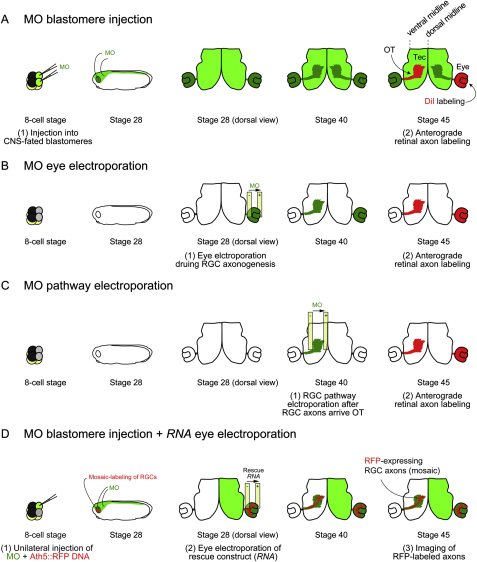
Experimental Schemes for Axon Degeneration Analysis, Related to Figure 5 (A) Injection into two dorsal animal blastomeres at 8-cell stage results in the delivery of MO into the CNS. At stage 45, one eye is labeled by DiI, which anterogradely travels to label RGC axons (optic tract: OT) reaching the contralateral optic tectum (Tec). (B) Electroporation into the eye primordium at stage 28 results in eye-specific MO delivery without affecting the brain. (C) Electroporation in the optic tectum at stage 40 results in the delivery of MO into the RGC axons without affecting their cell bodies in the contralateral eye. (D) Rescue experiment shown in Figure 6J. Ath5:RFP, a plasmid which drives RFP expression in RGCs, and MO are coinjected into one dorsal animal blastomere at 8-cell stage. This results in a unilateral CNS delivery of the MO and mosaic RFP-labeling of RGCs in the same side. The rescue construct is then electroporated into the same eye at stage 28, when RGC axonogenesis occurs. RFP-labeled RGC axons reaching the contralateral optic tectum are imaged at stage 45.

We next assessed retinal axons, in which *lb2* translation is blocked by MO, by lipophilic dye (DiI) labeling (Figures 5A and S5A). Although RGC axons in LB2 morphants appeared to be relatively normal when they reached their target in the optic tectum at stage 39/40, a severe axonal loss was observed 2 days later at stage 45 (Figure 5B). Approximately 70% of the brains had fewer than 15 DiI-labeled axons at stage 45, whereas less than 5% of control morphant brains did (Figure 5D). Degenerating axons, distinguished by their beaded and discontinuous morphology (Figure 5C), were evident along the pathway in LB2 morphant brains at stage 45, suggesting that LB2 may be required for axon maintenance. Morphants appeared normal with no overt differences in embryo morphology and DiI filling of the eye (data not shown). Furthermore, HRP filling of LB2 morphant eyes also showed a similar loss of retinal axons at stage 45 (data not shown), indicating that the reduction in the number of retinal axons is not due to differences in DiI uptake. Next, in order to rule out the possibility of nonspecific off-target effects of the MO (Heasman, 2002), we performed rescue experiments by coinjecting a MO-resistant LB2-GFP expression vector (Prüfert et al., 2004). LB2-GFP almost completely rescued the axon degeneration phenotype (Figures 5B and 5D), demonstrating that the phenotype is specific to the loss of LB2 function. We next asked whether this phenotype was a distinct axonal event, rather than a consequence of cell body death, which is predominantly mediated by the caspase-3 pathway (Nikolaev et al., 2009). Examination of cell body death using active caspase-3 as a marker showed no difference between CoMO- and LB2MO-injected embryos at stages 41–45 (Figures 5E and 5F).

**Figure 6:**
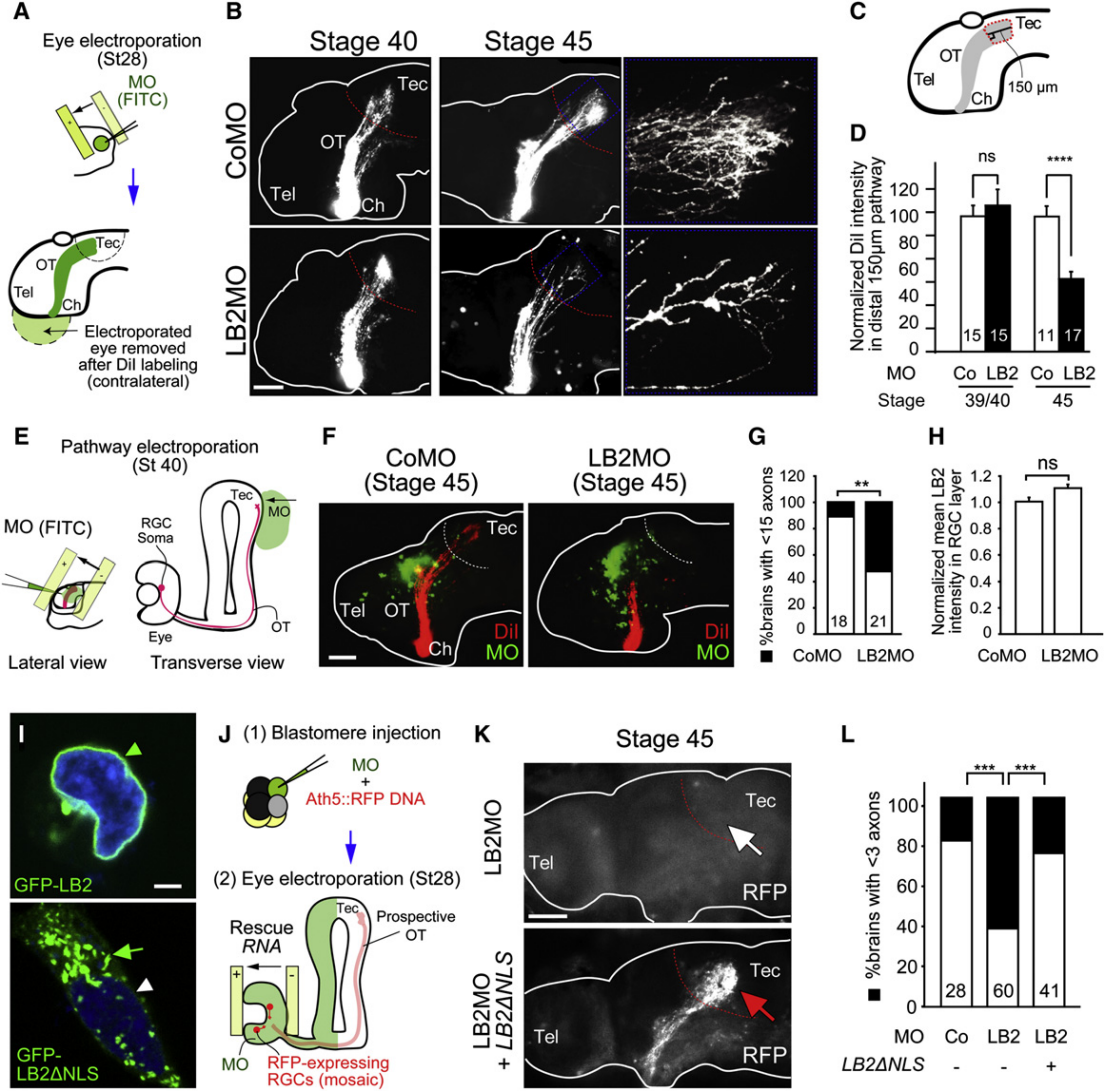
LB2MO-Induced Pathway Degeneration Is Mainly Axonal and Results from Inhibition of Local LB2 Synthesis (A) Eye electroporation. (B) DiI-labeled RGC axons in eye-electroporated embryos. (C and D) The mean DiI intensity of the distal 150 μm of the RGC pathway (mean ± SEM; n = no. of brains; 3 replicates; ^∗∗∗∗^p < 0.0001; Mann-Whitney). (E) Pathway electroporation. (F and G) DiI-labeled RGC axons in pathway-electroporated embryos (mean ± SEM; n = no. of brains; 3 replicates; ^∗∗^p < 0.01; Fisher's exact). (H) Nuclear LB2 immunofluorescent intensity in the RGC layer normalized to its intensity in the INL (mean ± SEM; for CoMO, total RGC no. = 1648, total INL cell no. = 2813, total no. of sections = 15; for LB2MO, total RGC no. = 1639, total INL cell no. = 3001, total no. of sections = 14; Mann-Whitney). (I) Wild-type LB2 and LB2ΔNLS localization in HEK293T cells (arrowhead: nuclear; arrow: cytoplasmic). (J) Rescue experiment. (K and L) Ath5:RFP-labeled RGC axons in LB2MO-injected embryos with or without LB2ΔNLS eye electroporation. Tel: telencephalon; Ch: optic chiasm; OT: optic tract; Tec: optic tectum. Scale bars: 65 μm in (B), (F), and (K) and 10 μm in (I). See also Figure S6.

**Figure S6:**
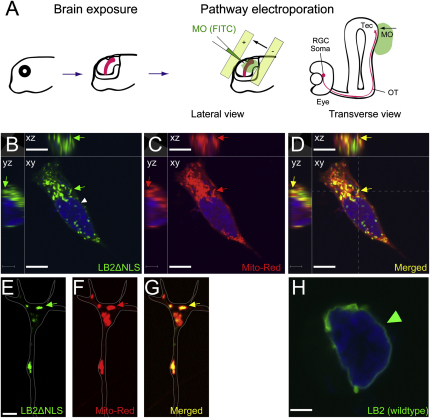
Axonally Synthesized LB2 Localizes to Mitochondria, Related to Figure 6 (A) Detailed schematic representation of pathway electroporation described in Figure 6E. At stage 40, one side of the optic tectum is exposed by removing the eye and skin before MO is electroporated. (B–D) HEK293T cells transfected with *Xenopus* LB2ΔNLS plasmid were labeled with MitoTracker, and imaged by a laser-scanning confocal microscope. LB2ΔNLS does not localize to the nuclear membrane (arrowhead) and instead localize to cytoplasmic structures that include mitochondria (arrow). Cross-sectional views seen from the dashed lines in (D) are shown left and above each image. (E–G) LB2ΔNLS also localizes to mitochondria in cultured *Xenopus* RGC axons (arrow). (H) In contrast, wild-type LB2 mainly localizes to the nuclear membrane. Scale bars: (B–D) and (H), 10 μm; (E–G), 5 μm.

Blastomere injection delivers the MO into the prospective brain as well as the eye (Figure S5A). To confirm the RGC-autonomous effect of LB2MO, we used targeted electroporation to deliver the MO specifically into the eye, without affecting the brain (Figures 6A and S5B). As with blastomere injection, the RGC axon projection was normal at stage 40 but became significantly less dense by stage 45 (Figure 6B), as quantified by the mean DiI signal intensity of the distal 150 μm portion of the projection (Figures 6C and 6D). A more central question, however, is whether LB2 synthesis in the axon is specifically required for axon survival. To inhibit *lb2* mRNA translation specifically in axons, we took advantage of the fact that distal RGC axons are anatomically compartmentalized away from the soma and navigate close to the lateral surface of the brain, making these axons easily accessible to electroporation (Chien et al., 1993; Falk et al., 2007; Holt, 1984). This allows delivery of LB2MO directly into distal axons (Figures 6E, 6F, S5C, and S6A), similar in principle to inhibition of axonal proteins using compartmentalized chambers in vitro (Hengst et al., 2009). We found that axonal delivery of LB2MO led to a significant axon loss (Figure 6G). Diffusion of the MO retrogradely into the soma was not observed at stage 45 in RGC bodies in the contralateral eye (absence of FITC-labeled MO; data not shown), and the nuclear LB2 intensity in the RGC layer was not reduced, ruling out the possibility of a nuclear LB2 knockdown by axonal LB2MO delivery (Figure 6H). To further examine whether axonally synthesized LB2 functions locally or in the nucleus after retrograde transport to the nucleus, we asked whether an LB2 lacking the NLS (LB2ΔNLS) could rescue the axon degeneration phenotype. This mutant form of LB2 does not enter the nucleus and instead localizes to cytoplasmic structures (Figure 6I). We introduced RNAs encoding LB2ΔNLS into RGCs by eye electroporation in LB2-depleted embryos that had been injected with a reporter plasmid to label RGCs with RFP (ath5::RFP) along with the LB2MO (Figures 6J and S5D). Strikingly, LB2ΔNLS almost completely rescued the LB2 loss-of-function phenotype of RGCs (Figures 6K and 6L), suggesting that extranuclear LB2 is sufficient for axon survival. Taken together, these results suggest that axonally synthesized LB2 is required and sufficient for RGC axon survival.

### LB2 Knockdown Leads to Mitochondrial Dysfunction

**Figure 7:**
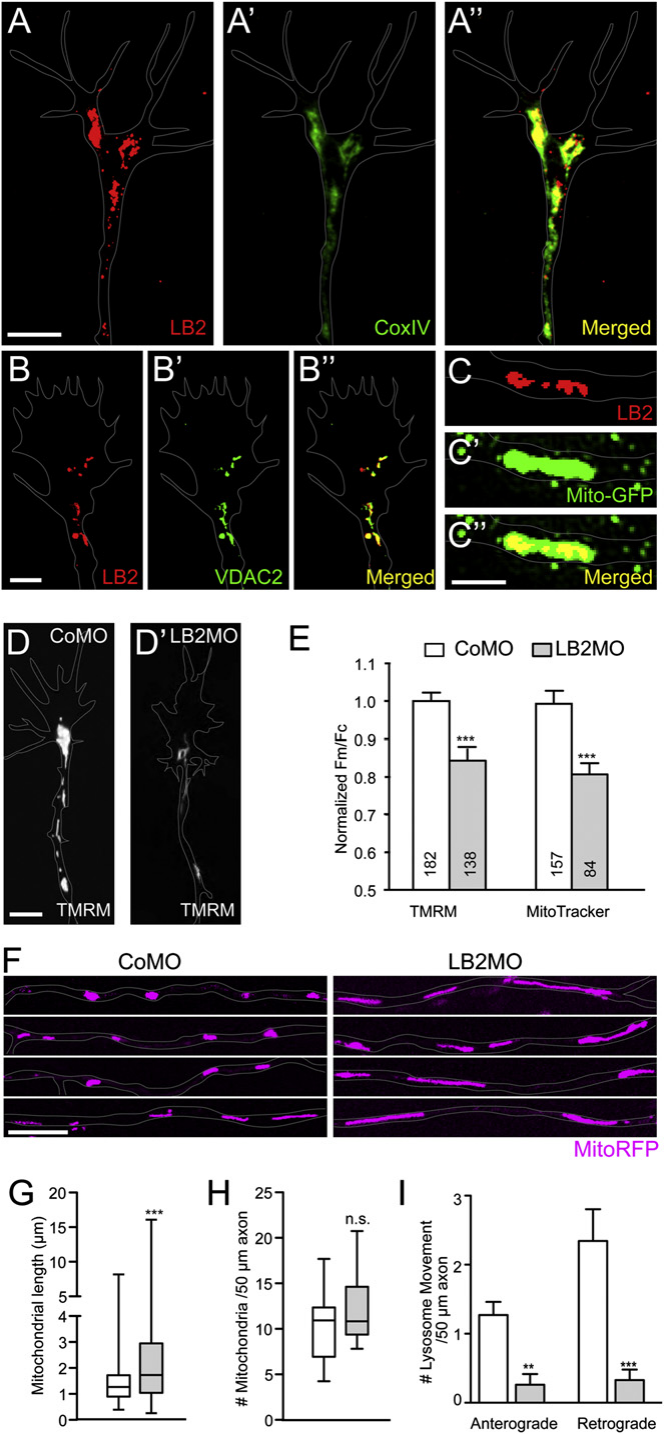
LB2 Knockdown Interferes with Mitochondrial Functions in Axons (A and B) LB2 coimmunostaining with CoxIV and VDAC2. (C) A single plane OMX super-resolution image of LB2 and Mito-GFP. (D) TMRM staining of CoMO- or LB2MO-injected axons and GCs. (E) Mitochondrial potential (Fm/Fc) in the distal 30 μm GCs/axons (mean ± SEM; n = no. of mitochondria analyzed; 3 replicates; ^∗∗∗^p < 0.0001; Mann-Whitney). (F and G) Mitochondrial length (box-and-whisker plot: minimum and maximum) (^∗∗∗^p < 0.0001; Mann-Whitney). (H) Mitochondrial number per unit length axon (box-and-whisker plot) (n.s.: not significant). (I) Anterograde and retrograde organelle transport measured by lysosome movements (mean ± SEM; 18 CoMO axons and 15 LB2MO axons; ^∗∗^p < 0.01 and ^∗∗∗^p < 0.001; Dunn's multiple comparison and Kruskal-Wallis). Scale bars: 1 μm in (C) and 5 μm in the rest. See also Figure S7.

**Figure S7:**
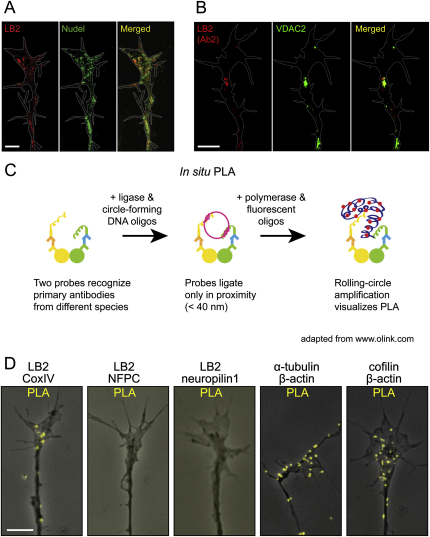
LB2 Localizes to Mitochondria, Related to Figure 7 (A) LB2 and Nudel show a relatively weak colocalization. (B) LB2 detected by a second LB2 antibody colocalizes with VDAC2, a mitochondrial protein. (C) Schematic representation of PLA technology. PLA signal from fluorescent oligonucleotides represents proximity of the two proteins of interest (within 40 nm). (D) Representative images of cultured RGC GCs, showing PLA signals obtained with indicated antibody pairs. LB2 interacts with CoxIV, but not with NFPC or neuropilin1. Positive controls (the last two panels) show specific signals within the GC, which is consistent with reported protein-protein interactions. Scale bars, 5 μm.

How does LB2 regulate axon survival? We concentrated on mitochondria because the mitochondrial proteome contains LB2 (Fountoulakis et al., 2002; Rezaul et al., 2005; Taylor et al., 2002), and mitochondrial dysfunction is strongly associated with axon degeneration in pathological conditions including multiple sclerosis (Su et al., 2009) and amyotrophic lateral sclerosis (Shi et al., 2010). Furthermore, maintenance of mitochondrial membrane potential and function is critical in caspase-3-independent neurite degeneration (Ikegami and Koike, 2003), a phenotype we observe in LB2 morphants (Figures 5B and 5E). Coimmunocytochemistry using antibodies against LB2 and CoxIV, a cytochrome *c* oxidase subunit localized to the inner mitochondrial membrane, revealed a significant overlap of LB2 and CoxIV in axons and GCs (Figure 7A: Pearson's coefficient = 0.800 ± 0.01, n = 7), which was confirmed by the proximity ligation assay (PLA) that produces a positive signal only when two different antibodies are within 40 nm (Figure S7C)—a distance consistent with a potentially direct molecular interaction (Fredriksson et al., 2002). LB2 also colocalizes with VDAC2, a mitochondrial outer membrane protein (Figures 7B and S7B), as well as with mitochondria-targeted GFP (Mito-GFP) (Figure 7C). OMX super-resolution microscopy analysis showed that LB2 localizes inside the mitochondria in a punctate pattern (Figure 7C). In line with these results, LB2ΔNLS that cannot be imported into the nucleus localizes to cytoplasmic structures that include mitochondria (Figures S6B–S6H). These results suggest that LB2 synthesized in distal axons where nuclear import machinery is not abundant may be imported into mitochondria.

We next examined how LB2 depletion affects mitochondria. First, we measured the mitochondrial membrane potential (ΔΨ_m_) using two indicators and found that it is significantly reduced in LB2-depleted axons (Figures 7D and 7E). We also analyzed mitochondrial morphology and found that LB2 morphant axons contained strikingly elongated mitochondria although the average number was not different from the control (Figures 7F–7H). Dynamic image analysis showed that the motile axonal mitochondria (approximately 20% of total) exhibited a slight but statistically nonsignificant decrease in motility in both directions in LB2-depleted axons (1.3 versus 1.1 anterograde movements/50 μm axon in 5 min, p = 0.14; 0.8 versus 0.4 retrograde movements/50 μm axon in 5 min, p = 0.13; n = 14 for CoMO and n = 17 for LB2MO; Mann-Whitney). A reduced mitochondrial potential and elongated morphology are associated with dysfunctional mitochondria (Ikegami and Koike, 2003; Yoon et al., 2006). Mitochondrial dysfunction impairs axonal transport of organelles such as lysosomes, which is a typical feature of degenerating axons (Kaasik et al., 2007), and causes optic neuropathies in humans (Carelli et al., 2004). Therefore, we analyzed the dynamics of lysosome movements in cultured retinal axons and found that LB2-depleted axons showed a significantly reduced number of directional movements (i.e., ≥3 μm movements occurring without a pause or a change of direction) compared to the control axons (Figure 7I). The reduction was observed in both anterograde and retrograde directions.

## Discussion

Here we have developed a proteomics strategy, DIGE-NCAT, to visualize subtle changes in the axonal proteome. The key advantage of proteomics-based approaches in detecting cue-induced mRNA translation and protein synthesis is the accumulation of signal throughout the cue stimulation period. Cue-induced local mRNA translation is a dynamic process regulated in a narrow time window. For example, the BDNF-induced increase in local mRNA translation activity in dendrites of cultured hippocampal neurons shows complex kinetics in which different parts of the same dendrite reach a peak at different times (Aakalu et al., 2001). Therefore, many mRNAs whose translation is induced by a cue may evade ribosome immunoprecipitation protocol (i.e., TRAP) performed at a fixed time point after the cue stimulation. The main advantage of DIGE-NCAT over other proteomics approaches is its sensitivity. We were able to detect subtle changes in axonal protein levels, both up and down, suggesting that this technique may prove to be a versatile tool to study dynamic changes of the axonal proteome including translational repression and/or protein degradation. Metabolic labeling represents another sensitive technique, but the use of nonradioactive material and an internal standard for more reliable comparison between multiple conditions give DIGE-NCAT more advantages.

This proteomics approach revealed, unexpectedly, that LB2 is axonally synthesized in response to extrinsic cue stimulation. LB2, a known nuclear protein, has previously been shown to be involved in regulating nuclear translocation and neuronal migration. In *Drosophila*, LB2 regulates the apical migration of nuclei in differentiating photoreceptors (R cells) by connecting the microtubule-organizing center (MTOC) to the nucleus to regulate nuclear translocation, a critical process for neuronal nuclear migration (Patterson et al., 2004). A recent study showed that *lb2* knockout mice exhibit specific defects in neuronal migration in the cerebral cortex and cerebellum (Coffinier et al., 2010), suggesting that LB2's role in regulating nuclear translocation may be evolutionarily conserved. However, our finding that LB2 is synthesized in distal axons shows that LB2 may also have an additional extranuclear role.

Consistent with our proteomics result, *lb2* mRNA localizes to axons and GCs in culture and, importantly, associates with translating ribosomes in retinal axons in vivo. Knockdown of *lb2* mRNA translation in axons caused retinal axon degeneration in vivo. One obvious possibility is that locally synthesized LB2 is transported back into the nucleus where it modulates nuclear activity required for axon stability, similar to how axonally synthesized CREB promotes axon survival (Cox et al., 2008). However, two pieces of evidence suggest that the accumulation and local function of axonally synthesized LB2 are crucial for axon survival. First, axon-specific delivery of LB2MO in vivo resulted in axon degeneration in 2 days, whereas no detectable changes in the LB2 protein level were observed in the RGC bodies. Interestingly, depletion of axonal LB2 resulted in axon degeneration without inducing caspase-3-dependent apoptosis in the cell bodies. In mice, misprojecting RGC axons degenerate through the caspase-6 pathway, which is activated by deprivation of trophic factors secreted from the optic tectum (Nikolaev et al., 2009); therefore, it will be interesting to know whether LB2MO-induced RGC axon degeneration is mediated by caspase-6 and whether trophic factor deprivation reduces axonal LB2. Second, LB2ΔNLS, an LB2 mutant that is not imported into the nucleus, could rescue the axon degeneration phenotype caused by LB2 depletion. Indeed, cytoplasmic localization of lamins was observed in rat cerebellar Purkinje cells in vivo (Toesca, 1992), and microarray analyses have identified mRNAs encoding lamins in embryonic mouse RGC axonal GCs (Zivraj et al., 2010), embryonic mouse DRG neuronal axons (Gumy et al., 2011), and axons of mature mammalian cortical neurons after axotomy (Taylor et al., 2009), suggesting that axonal synthesis of lamins may be utilized in diverse developing and regenerating axons.

A recent study showed a direct interaction between lamin B and Nudel (Ma et al., 2009), providing a possible mechanism for LB2's actions in the cytoplasm. Nudel forms a complex with Lis1/dynein/dynactin in the cytoplasm to regulate cargo by utilizing ATP, and disrupting the binding of Nudel to the dynein motor complex impairs retrograde axonal transport (Liang et al., 2004; Nguyen et al., 2004; Sasaki et al., 2000; Zhang et al., 2009). Because defective retrograde transport is strongly associated with axon degeneration (Jing and Malicki, 2009), one plausible cytoplasmic role for LB2 would be direct regulation of axonal transport through its interaction with Nudel. We did not, however, detect a strong colocalization between LB2 and Nudel in axons and GCs (Figure S7A: Pearson's coefficient 0.417 ± 0.03, n = 5), and we observed a generally decreased cargo transport activity instead of a specific defect in retrograde transport (Figure 7I). Nonetheless, the possibility that extranuclear LB2 interacts with Nudel to regulate axonal cargo transport should not be excluded.

A general reduction in axonal transport prompted us to investigate mitochondria, the main source of ATP in axons, and our data indicate that axonally synthesized LB2 functions in mitochondria to support axon survival. The local synthesis of LB2 in a distal process remote from the nucleus may enable the protein to escape nuclear localization and take on cytoplasmic functions. Indeed, we show that deleting the 6 amino acid NLS at the C terminus enhances the mitochondrial targeting of LB2 (Figures S6B–S6G). Intriguingly, MitoProt, Software that predicts mitochondrial proteins with high accuracy (Claros and Vincens, 1996), shows that *Xenopus* LB2 has an extremely high probability of mitochondrial localization (p = 0.97). Other nuclear-encoded mitochondrial proteins such as CoxIV, Cox17, CoQ7, and Hsp70 are also locally synthesized in the presynaptic nerve terminal (Aschrafi et al., 2010; Gioio et al., 2001), and we have identified such proteins in this proteomics study (Table S1), suggesting that axonal mitochondria require a local supply of new proteins. It will be interesting in the future to investigate whether *lb2* mRNA contains an axonal localization element as recently shown for *coxIV* mRNA (Aschrafi et al., 2010). Exactly how LB2 modulates mitochondrial function still remains to be elucidated. Lamins regulate transcription (Spann et al., 2002), protein import (Busch et al., 2009), and morphology (Dahl et al., 2004) in the nucleus, and LB2 might play similar roles in mitochondria. Another interesting possibility is that LB2 controls mitochondrial fission. Downregulation of mitochondrial fission is associated with senescence and optic atrophy (Waterham et al., 2007; Yoon et al., 2006) and results in elongated mitochondria, a phenotype we observed in LB2-depleted axons (Figures 7F and 7G).

Mitochondrial function is essential for numerous processes required to maintain axonal health and stability (Knott et al., 2008), and accumulating evidence points to mitochondrial dysfunction as one of the main causes in some neurodegenerative diseases, including familial Parkinson's disease (Lin and Beal, 2006) and LHON (Wallace et al., 1988). Our data showing that LB2 depletion causes a reduction in mitochondrial membrane potential (Figures 7D and 7E) suggest an intriguing model whereby extrinsic cues secreted from the optic tectum, such as En-1, stimulate in distal RGC axons LB2 synthesis, which then acts locally to regulate mitochondrial function in order to meet high metabolic demands of these axons. Intriguingly, a recent study showed that En-1 also stimulates translation of mRNAs encoding mitochondrial complex I subunits and protects against degeneration of midbrain dopaminergic neurons in an animal model of Parkinson's disease (Alvarez-Fischer et al., 2011), suggesting that the translational control of mRNAs encoding nuclear-encoded mitochondrial proteins in axons is a conserved mechanism to regulate axonal survival.

In summary, the results presented here suggest that LB2 is an axonally synthesized protein with an extranuclear role in mitochondria that is necessary to maintain the integrity of distal axons. Our findings may provide insight into the mechanisms underlying the cell type-specific nature of some laminopathies.

## Experimental Procedures

### Embryos and Blastomere Injection

*Xenopus laevis* embryos were fertilized and raised as described (Drinjakovic et al., 2010). EGFP-L10a, EGFP-LB2, and Mito-XFP constructs were kind gifts from N. Heintz (Rockefeller University), G. Krohne (University of Würzburg), and M. Coleman (Babraham Institute), respectively. Ath5:RFP was previously described (Zolessi et al., 2006). LB2ΔNLS was made by inverse PCR from EGFP-LB2. Capped RNAs were made using mMessagemMachine SP6 kit (Ambion). Translation blocking MOs were designed and supplied by GeneTools (Philomath, OR, USA). Embryos were injected at the 8-cell stage with MO, DNA, or RNA in dorsal animal blastomeres (presumptive CNS) as described previously (Drinjakovic et al., 2010).

### Electroporation

Eye electroporation was performed as described (Falk et al., 2007). For pathway electroporation, the lateral side of the brain at stage 40 was exposed, and MO was electroporated close to the lateral side of the brain.

### RGC Pathway Visualization and Analysis

DiI labeling of RGC axons was performed as described (Drinjakovic et al., 2010). DiI intensities were measured using Openlab (Improvision). Injections of ath5:RFP (200 pg per blastomere) resulted in a mosaic expression of RFP in RGCs, which labeled fewer retinal axons compared to the DiI method. For this reason, the brains with fewer than 15 and 3 axons were counted when quantitating the axon degeneration phenotype by DiI and ath5:RFP labeling, respectively.

### TRAP

Eye transplantation (Holt, 1984) and immunoprecipitation (Heiman et al., 2008) were performed as described. One nanogram of GFP-L10a or GFP RNA (negative control) was delivered by blastomere injections. Ten pooled brain hemispheres were used per group, and the presence of candidate mRNAs was examined by RT-PCR.

### Retinal/Axon Cultures and Stimulation

For all the retinal culture experiments, eye primordia from stage 35/36 embryos were dissected and cultured at 20°C for 40 hr as described (Drinjakovic et al., 2010). This stage corresponds to a stage between 42 and 43 in vivo, which is approximately 1–2 days prior to stage 45 when the LB2MO-induced axon degeneration phenotype appears. External cues used are provided in the Extended Experimental Procedures. Axon cultures were prepared as described (Campbell and Holt, 2001) by separating distal axons from the eye and removing eyes/loose cells.

### Mitochondrial Membrane Potential (ΔΨ_m_) Measurement

Live retinal cultures were incubated with 20 nM tetramethylrhodamine, methyl ester (TMRM), and MitoTracker red CM-H2XRos (MitoTracker) for 20 min and washed with culture medium three and six times, respectively. ΔΨ_m_ was expressed by fluorescence ratio of mitochondria (Fm) to cytoplasm (Fc) calculated using Openlab. Fc was measured no more than 3 μm away from the mitochondrion of interest.

### PCR and RT-PCR

RNA-only and DNA/RNA copurification were performed using RNeasy micro kit (QIAGEN) and DNeasy blood and tissue kit (QIAGEN), respectively. For quantitative RT-PCR, cDNA was prepared from total RNA extracted from stage 41 embryonic eyes and amplified on a Rotor-Gene 3000 (Corbett) using QIAGEN kits.

### ISH

ISH on retinal sections and FISH of cultured axons were performed using digoxigenin (DIG)-labeled riboprobes generated from IMAGE clones as previously described (Zivraj et al., 2010).

### Immunofluorescence

Immunostaining, imaging and analysis were performed as described (Piper et al., 2006). Pearson's coefficients were calculated using Volocity (Perkin Elmer). For experiments shown in Figure 6I, HEK293T cells were transfected with LB2ΔNLS plasmid using Lipofectamine 2000 (Invitrogen), labeled by MitoTracker, fixed, and imaged using a laser-scanning confocal microscope (Leica) and a 60× 1.4NA oil immersion objective (Leica). Optical sections at 0.3–1.0 μm separation were taken and analyzed using Volocity. Immunostaining of sections was performed as described (Drinjakovic et al., 2010). For LB2 staining of embryonic sections, unfixed embryos were anesthetized, embedded in OCT, rapidly frozen in liquid nitrogen, sectioned by a cryostat, and then fixed in methanol. CellProfiler (Broad Institute) was used for quantification of nuclear LB2. For OMX imaging, retinal cultures obtained from Mito-GFP (200 pg per blastomere) injected embryos were fixed, immunostained for LB2, and imaged using DeltaVision OMX 3D-SIM System V3 controlled by softWoRx 5.0.0 (Applied Precision) and a 100× 1.4NA oil objective.

### Live Imaging

Cultured axons were imaged under a Perkin Elmer Spinning Disk UltraVIEW ERS, Olympus IX81 Inverted microscope, and a 60× 1.2NA water immersion objective. Mitochondria were visualized by blastomere injection of Mito-RFP plasmid (200 pg per blastomere), and lysosomes were labeled with Lysotracker Red (Invitrogen). Images were taken for 5 min at a 1 to 3 s interval and analyzed using Volocity.

### DIGE-NCAT

AHA (Invitrogen) (50 nM and 500 nM for retinal eye cultures and DIGE-NCAT, respectively) containing samples were labeled using Click-iT TAMRA kit (Invitrogen), gel-separated, fixed, and visualized using a TyphoonTM 9410 imager (GE Healthcare). The total lane intensity in 1D gel was measured using ImageJ (NIH). For DIGE-NCAT experiments, 1,000 eyes were used per condition for axon-only culture. Some of the eyes were lysed and labeled with Cy5 CyDye (Minimal labeling kit: GE Healthcare), and the rest of the eyes were lysed without labeling. Axon-only cultures were stimulated with AHA/cue, RNA and protein were copurified using PARIS (Ambion), and RNA concentrations were used to equilibrate samples. The protein lysate was then labeled with TAMRA, mixed with 50 μg of CyDye-labeled + 150 μg of unlabeled eye lysates, 2D-DIGE separated, visualized, and analyzed by biological variation analysis. Cy5 was used as a standard for multigel comparison. MS analyses were performed at the Cambridge Centre for Proteomics (University of Cambridge) and Alphalyse A/S (Denmark).

Extended Experimental Procedures **Embryos**

*Xenopus laevis* embryos were prepared by in vitro fertilization, raised in 0.1X Modified Barth's Saline (MBS) at 14°C –25°C, and staged according to Nieuwkoop and Faber (Nieuwkoop and Faber, 1967).

**Capped mRNA Synthesis and Morpholinos**

L10a cloned into pEGFP-C2 (Clontech) (GFP-L10a) was a kind gift from Nathaniel Heintz (Rockefeller university, New York, NY). In order to make RNA, an SP6 promoter was inserted at NheI/AgeI sites. GFP-L10a RNA was made from a DraIII-linearized plasmid by in vitro transcription using mMessagemMachine SP6 kit (Ambion) according to manufacturer's instructions. EGFP-laminB2 was a kind gift from Georg Krohne (University of Würzburg, Germany). Capped mRNA was prepared using the same protocol as GFP-L10a RNA except that the plasmid was linearized with MluI instead of DraIII. LB2ΔNLS was made by inverse PCR from EGFP-laminB2 using the following primers: 5′- GAAGAGGAATATGAGGAAGGT −3′ and 5′- TCTAGAGGTACGGGTGGCA −3′. Mitochondrially targeted GFP and RFP plasmids (Mito-GFP and Mito-RFP) were kind gifts from Michael Coleman (Babraham Institute, UK). Ath5:RFP was previously described (Zolessi et al., 2006). Antisense lamin B2 MO (LB2MO) and control MO (CoMO) conjugated to FITC at the 3′ end were designed and supplied by GeneTools (Philomath, OR, USA): LB2MO, 5′-ACCGACTTGGTGTAGCAGTAGCCAT-3′; CoMO, 5′-CCTCTTACCTCAGTTACAATTTATA-3′.

**Blastomere Injection and Electroporation**

Embryos were injected at the 8-cell stage in dorsal animal blastomeres as described previously (Drinjakovic et al., 2010). 0.63–1.25 pmol of LB2MO and 1.25 pmol of CoMO were injected per blastomere. For rescue, 1.25 pmol of LB2MO was injected with 1 ng of LB2-GFP mRNA.

Eye electroporation was performed as described (Falk et al., 2007) using one set of 8 electric pulses (50 ms long, 1000 ms intervals) delivered at 18V. For pathway electroporation, the lateral side of the brain at stage 40 was exposed as described (Chien et al., 1993) and positioned between the two electrodes. Two sets of 8 electric pulses (50 ms long, 1000 m intervals) were delivered at 18V. Ten nl of 1.35 mM morpholino solution was pulsed 5–6 times close to the lateral brain. The embryos were recovered in 0.25X MBS + 0.75% antibiotics (PSF; GIBCO) and 0.02% of MS-222 (Sigma) for at least 6 hr. After recovery, the embryos were transferred to 0.1X MBS with 1% PSF until stage 45.

**RGC Pathway Visualization and Analysis**

For DiI labeling of retinal axons, embryos were first fixed in 4% paraformaldehyde (PFA) dissolved in 1X PBS at desired stages. The lens was removed, and the eye cavity was filled with DiI (Invitrogen) dissolved in chloroform. After 2–3 days, the brain was dissected out, mounted, and visualized. For quantification of the DiI intensities, the lateral, middle, and medial images of the pathway were merged. The distal 150 μm of the pathway was delineated using the most dorsal, ventral, and distal pathway edges as borders. Mean intensity was measured using Openlab (Improvision). Statistical analyses for pathway intensities as well as all the other experiments were performed using InStat (GraphPad). Injections of ath5:RFP (200 pg per blastomere) resulted in a mosaic expression of RFP in RGCs, which labeled fewer retinal axons compared to the DiI method. For this reason, the brains with fewer than 15 and fewer than 3 axons were counted when quantitating the axon degeneration phenotype by DiI and ath5:RFP labeling, respectively.

**TRAP**

The TRAP experiment was performed as explained in Figure 4D. Eye transplantation (Holt, 1984) and immunoprecipitation (Heiman et al., 2008) were performed as described previously. Briefly, 1 ng of GFP-L10a or GFP RNA (negative control) was injected into the two dorsal animal blastomeres (presumptive CNS) at the 8-cell stage. At stage 26 (eye vesicle stage), the left eye of an injected donor embryo was grafted to an uninjected host embryo of the same stage. At stage 41 (target-arriving stage), the contralateral brain hemisphere containing the axons of the transplanted eye was dissected out. GFP-L10a-containing ribosome-mRNA complexes were immunoprecipitated from ten pooled brain hemispheres, from which RNAs were isolated using Trizol (Invitrogen). Isolated RNA was reverse-transcribed by using SuperScript III reverse transcriptase (Invitrogen), and the presence of candidate mRNAs was examined by PCR.

**Retinal/Axon Cultures and Stimulation**

For all the retinal culture experiments, eye primordia from stage 35/36 embryos were dissected and cultured at 20°C for 40 hr as described (Drinjakovic et al., 2010). This stage corresponds to a stage between 42 and 43 in vivo, which is approximately 1–2 days prior to stage 45 when the LB2MO-induced axon degeneration phenotype appears. External cues used include: BDNF (200 ng/ml; Sigma), fetal bovine serum (GIBCO; HI = heat inactivated, DIA = dialyzed), En-1 (150 ng/ml; kind gift from Alain Prochiantz, CNRS, France), Sema3A (800 ng/ml; R&D Systems), netrin-1 (600 ng/ml—homemade; 1 μg/ml—R&D Systems), and *Xenopus* yolk extract (Homemade). En-1 was dialyzed on nitrocellulose filtration membrane (Millipore) against 1X PBS for 20 min at 4°C. Axon-only cultures were prepared as described (Campbell and Holt, 2001) by separating distal axons from the eye and removing eyes/loose cells.

**Mitochondrial Membrane Potential Measurement**

For ΔΨ_m_ measurements, live retinal cultures were incubated with 20 nM TMRM or MitoTracker (Invitrogen; resuspended in dimethylsulfoxide, DMSO) for 20 min and washed with culture medium 3 or 6 times, respectively. Fm was measured by calculating the mean intensity of individually distinguishable mitochondria in the distal 30 μm of GCs/axons using Openlab. Fc was similarly measured no more than 3 μm away from the mitochondrion of interest. The Fm/Fc ratios were then normalized to control.

**PCR and RT-PCR**

RNA-only and DNA/RNA copurification were performed using RNeasy micro kit (QIAGEN) and DNeasy blood and tissue kit (QIAGEN), respectively. For quantitative RT-PCR, cDNA was prepared from total RNA extracted from stage 41 embryonic eyes and amplified on a Rotor-Gene 3000 (Corbett Life Science) using QIAGEN kits. The following primers were used: for *lamin A*, 5′- AGTCCCCCCAGTGATTTAGTGTGG-3′ and 5′- TCGCACCAGTTTCCTCATAGCAAC-3′; for *lamin B1*, 5′- GCTCATTCTGCCTCTGCCACTG-3′ and 5′- AGTTCCCAACCCCCCAAAGG-3′; for *lamin B2* (Figure 3A), 5′-GTTTGGCGGGAATATCTAACCGC-3′ and 5′-GTTAAGCTCGTCAAGATCTGATCG-3′; for *lamin B2* (Figure 4), 5′-AAAAGATGCTGACCTGT-CCACGG-3′ and 5′-CCTCTGCCTCCAAACTACGCTTCTC-3′; for *brn3*, 5′- TGAGCGATTCAA-GCAGAGGAGG-3′ and 5′- TGCGACAGGGTGAGGGATTCAAAC-3′; for *pax6*, 5′- AACGGACAAACTGGCACTTGGG-3′ and 5′- CCTCGTCTGAGTCTTCACCATTGG-3′; and for *β-actin*, 5′-CGTAAGGACCTCTATGCCAA-3′ and 5′-TGCATTGATGACCATACAGTG-3′.

**ISH on Retinal Cryosections and Cultures**

DIG-labeled riboprobes were generated from the following IMAGE clones as previously described (Blichenberg et al., 1999): for *lamin A*, clone 8825513; for *lamin B1*, clone 4684248; and *lamin B2*, clone 5157193. *Pax6* probe was previously described (Hirsch and Harris, 1997). FISH of cultured retinal GCs was performed as described (Blichenberg et al., 1999). ISH on retinal cryosections was performed as described (Mann et al., 2002).

**Immunofluorescence**

Immunostaining, imaging, and analysis were performed as described (Piper et al., 2006) using the following antibodies: anti-LB2 antibodies (Ab1—mouse monoclonal clone LN43, 1:100, Abcam; Ab2—rabbit polyclonal, 1:200, ab8983, Abcam; Ab3- mouse monoclonal clone X223, 1:100, Santa Cruz Biotechnology); rabbit polyclonal anti-CoxIV antibody (1:100, Abcam); goat polyclonal VDAC2 antibody (1:100, Abcam); and mouse monoclonal β-actin antibody (1:400, Abcam). Samples were fixed for 3 min with 2% paraformaldehyde/7.5% sucrose in PBS, washed in PBS, and then further fixed and permeabilized with methanol at −20°C for 5 min prior to immunostaining. Primary antibodies were incubated overnight at 4°C and secondary antibodies conjugated to Alex Fluor dyes (Invitrogen) were incubated for 1 hr at room temperature (RT). Imaging and analysis for quantitative immunofluorescence (QIF) experiments were performed blind to the experimental condition. Noncollapsed GCs were imaged using a Nikon Optiphot inverted microscope equipped with a 100x 1.4NA oil-immersion objective and a CCD camera (Hamamatsu). GCs were chosen randomly by using phase optics to avoid biased selection of fluorescence, and then a fluorescent image was captured, exposure time being kept constant and below greyscale pixel saturation. For quantitation of fluorescence intensity, the outline of a GC was manually drawn on a phase-contrast image by using the “freehand tool” in OpenLab software (Improvision) and defined as the region of interest (ROI). The growth cone central domain and lamellipodia, but not fillopodia, were included. The same ROI was then superimposed onto the matching fluorescent image, and the sum of fluorescent intensity within the ROI was divided by its area to generate the mean fluorescent pixel intensity per unit area (MPI) (i.e., protein level) using the “measurements” tool in OpenLab. The GC outline was then placed in an adjacent area clear of cellular material to record the background fluorescent intensity. This reading was subtracted from the GC reading, yielding the background-corrected intensity. The background-corrected MPIs and areas were collected from 38 to 97 GCs per group, and compared between groups by unpaired t test (Figures 3C and S3M) or one-way ANOVA followed by Bonferroni's post hoc multiple comparison tests (Figures 4B and 4C). p values lower than 0.05 were regarded as statistically significant. Data were normalized to the control groups and presented as mean ± standard error of the mean (SEM). Samples from independent experiments were stained, imaged, and processed under identical conditions. Counts in which the experimenter was blind to the identity of samples resulted in similar results. For colocalization, Pearson's coefficients were calculated using Volocity Software (Perkin Elmer). For experiments shown in Figures 6I and S6B–S6H, HEK293T cells were transfected with LB2ΔNLS plasmid by using Lipofectamine 2000 (Invitrogen) according to the manufacturer's instructions. Transfected cells were then plated on a poly-L-Lysine-coated coverslip, labeled by MitoTracker-Red, fixed with 4% paraformaldehyde, and imaged using a laser scanning confocal microscope (Leica) and a 60X 1.4NA oil immersion objective (Leica). Optical sections at 0.3–1.0 μm separation were taken and analyzed in Volocity (Perkin Elmer). Immunostaining of sections was performed with transverse sections of embryos fixed in 4% PFA, treated with 30% sucrose, and embedded in OCT, using activated caspase-3 antibody (1:500; BD Biosciences), neurofilament antibody (1:500; clone 3A10, Developmental Studies Hybridoma Bank), or Brn3 antibody (1:500, Santa Cruz). For LB2 staining of embryonic sections, unfixed embryos were anesthetized, embedded in OCT, rapidly frozen in liquid nitrogen, sectioned by a cryostat, and then fixed in methanol. CellProfiler (Broad Institute) was used for quantification of nuclear LB2.

**OMX Microscopy**

Retinal cultures obtained from Mito-GFP (200 pg per blastomere) injected embryos were fixed, immunostained for LB2, and imaged using DeltaVision OMX 3D-SIM System V3 (Applied Precision). All data capture used an Olympus 100x 1.4NA oil objective, 405 nm, 488 nm, and 593 nm laser illumination and standard excitation and emission filter sets. 3D-SIM images were sectioned using 125 nm z-step size. Raw 3-phase images were rendered and reconstructed in 3D by softWoRx 5.0.0 (Applied Precision) Software.

**Live Imaging**

Cultured axons were imaged using a Perkin Elmer Spinning Disk UltraVIEW ERS, Olympus IX81 Inverted microscope and a 60X 1.2NA water immersion objective. Mitochondria were visualized by blastomere injection of Mito-RFP plasmid (200 pg per blastomere), and lysosomes were labeled with Lysotracker Red (Invitrogen) according to the manufacturer's instructions. Images were taken for 5 min at a 1 to 3 s interval and analyzed with Volocity Software (Perkin Elmer).

**In Situ PLA**

Duolink in situ PLA (Olink Bioscience) was performed on cultured retinal explants from *Xenopus* embryos according to manufacturer's recommendations. The following primary antibody pairs were used: mouse anti-laminB2 (1:100, Abcam) and rabbit anti-CoxIV (1:100, Abcam); mouse anti-laminB2 and rabbit anti-NFPC (1:500; a kind gift from Roger Bradley, Montana State University) (Heggem and Bradley, 2003); mouse anti-laminB2 and rabbit anti-neuropilin1 (1:200, Zymed); mouse anti-β-actin (1:500, Abcam) and rabbit anti-α-tubulin (1:500, Abcam); and mouse anti-β-actin and rabbit-anti-cofilin (1:1000, Abcam). Briefly, cultures were fixed for 3 min with 2% paraformaldehyde/7.5% sucrose in PBS, washed in PBS, and then further fixed and permeabilized with methanol at −20°C for 5 min or fixed for 1 hr with 2% paraformaldehyde/7.5% sucrose in PBS, washed in PBS, and then permeabilized with 0.1% Triton X-100 for 3 min. Cultures were washed again in PBS, blocked for 1 hr at room temperature in 5% heat-inactivated goat serum in PBS, and then incubated overnight with indicated antibody pairs diluted in blocking solution. The following day, PLA was performed based on the manufacturer's instructions, using blocking solution for PLA probe dilution and supplied diluent for ligation and amplification steps. All washes preceding the ligation step used PBS. Subsequent to the ligation step, supplied wash buffers were used according to manufacturer's instructions. Cultures were mounted with supplied Duolink mounting medium.

**Western Blot Analysis**

Western blot experiments were carried out as described (Drinjakovic et al., 2010) using antibodies against LB2 (1:1000) or α-tubulin (1:3000; Abcam).

**2D-DIGE**

2D-DIGE experiments were performed as described (Casasoli et al., 2008) using 50 μg of the retinal culture lysate per condition. For second dimension separation, nonlinear IPG strips (13 cm long, pH3-10; GE Healthcare) and 10% w/v acrylamide gel were used. The standard lysate was prepared by pooling fractions of lysates from all experimental conditions into one sample. Each sample was labeled with Cy3 CyDye (GE Healthcare) and coseparated with Cy5 CyDye-labeled standard (GE Healthcare). The 2D gels were analyzed using the biological variation analysis (BVA) for multi-gel comparison as described (Casasoli et al., 2008). 2D gels were fixed and silver-stained using the protocol from the Cambridge Centre for Proteomics (CCP; University of Cambridge, UK; http://www.bio.cam.ac.uk/proteomics).

**AHA and DIGE-NCAT**

AHA (Invitrogen) dissolved in DMSO was used at 50 nM and 500 nM concentrations for retinal eye cultures and DIGE-NCAT, respectively. AHA-labeled samples were labeled using Click-iT Tetramethylrhodamine (TAMRA) protein analysis detection kit (Invitrogen), gel-separated, fixed, and visualized using a TyphoonTM 9410 imager (GE Healthcare). 1D gels were Coomassie stained using the protocol provided by CCP, and the total lane intensity was measured using ImageJ (National Institutes of Health).

For DIGE-NCAT experiments, 1,000 eyes from stage 37–39 embryos were cultured per condition for 28 hr. Axons were severed, separated eyes were collected, and any loose cells were scraped off. Some of the eyes were lysed and labeled with Cy5 CyDye according to the manufacturer's protocol (Minimal labeling kit: GE Healthcare), and the rest of the eyes were lysed without any labeling. Axon-only cultures were stimulated with AHA/cue, and RNA and protein were copurified using the PARIS kit (Ambion). The total RNA concentration was measured using Nanodrop and was used to equilibrate the protein concentration. The protein lysate was then labeled with TAMRA, mixed with 50 μg of CyDye-labeled + 150 μg of unlabeled eye lysates, separated, visualized, and analyzed using 2D-DIGE and BVA. The Cy5 CyDye labeled eye lysate was used as a standard for multigel comparison. The gels were then fixed and silver-stained using the protocol from CCP.

**MS Analysis**

MS analyses were performed by CCP (Figure S1) and Alphalyse A/S (Table S1: http://www.alphalyse.com). Details on MS methods can be found on their websites.

## Supplemental Information

Table S1. MS Identification of AHA-Labeled Spots from DIGE-NCAT Experiment, Related to Figure 2 Twelve axonally synthesized proteins were identified by MS. These included proteins whose axonal synthesis was increased (↑), decreased (↓), or unchanged (=) by En-1 stimulation. Molecular weights (MW) are represented in Daltons, and the score represents the MASCOT score. Spot numbers correspond to those in Figure S2H.

Document S1. Article plus Supplemental Information
