# Supplementary material for: Local Translation of Extranuclear Lamin B Promotes Axon Maintenance
Source: Cell. 2012 Feb 17;148(4):752–64. doi: 10.1016/j.cell.2011.11.064 (PMC3314965; doi:10.1016/j.cell.2011.11.064)
Supplement: Table S1. MS Identification of AHA-Labeled Spots from DIGE-NCAT Experiment, Related to Figure 2 — Twelve axonally synthesized proteins were identified by MS. These included proteins whose axonal synthesis was increased (↑), decreased (↓), or unchanged (=) by En-1 stimulation. Molecular weights (MW) are represented in Daltons, and the score represents the MASCOT score. Spot numbers correspond to those in Figure S2H. [file mmc1.pdf]

Table S1. MS Identification of AHA-Labeled Spots from DIGE-NCAT Experiment, Related to Figure 2

Sample	Protein ID	GI number	MW	Score	Sequence coverage	Changed by En-1
1	lamin B2	gi|147901703	71218	254	51%	↑
2	lamin B2	gi|147901703	71218	351	57%	↑
3	ATP synthase, H+ transporting, mitochondrial F1 complex, alpha subunit, isoform 1	gi|148224211	59911	94	23%	↓
4	heterogeneous nuclear ribonucleoprotein K	gi|147903924	43913	150	41%	↑
5	heat shock 70kDa protein 1-like	gi|148222597	71144	106	22%	↓
6	mitochondrial ATP synthase beta subunit	gi|148223359	56338	127	32%	↓
7	actin, cytoplasmic type 8	gi|148222128	41777	485	54%	=
8	creatine kinase, mitochondrial 1B	gi|147900608	46953	319	23%	=
9	creatine kinase, mitochondrial 1B	gi|147900608	46953	319	23%	↑
10	aldo-keto reductase family 1, member B1 (aldose reductase)	gi|147905319	36041	123	37%	↑
11	Gapd-prov protein	gi|27882192	35812	282	40%	↑
12	heterogeneous nuclear ribonucleoprotein D-like-B	gi|148235301	32935	240	35%	↓
